# Interleukin-17-Producing CD4^+^ T Cells Promote Inflammatory Response and Foster Disease Progression in Hyperlipidemic Patients and Atherosclerotic Mice

**DOI:** 10.3389/fcvm.2021.667768

**Published:** 2021-04-26

**Authors:** Yin Wang, Wenming Li, Tingrui Zhao, Yao Zou, Tao Deng, Zhangyou Yang, Zhiyi Yuan, Limei Ma, Ruihong Yu, Tingting Wang, Chao Yu

**Affiliations:** ^1^College of Pharmacy, Chongqing Medical University, Chongqing, China; ^2^Chongqing Key Laboratory for Pharmaceutical Metabolism Research, Chongqing, China; ^3^Chongqing Pharmacodynamic Evaluation Engineering Technology Research Center, Chongqing, China; ^4^Department of Clinical Laboratory, University-Town Hospital of Chongqing Medical University, Chongqing, China; ^5^Research Center of Pharmaceutical Preparations and Nanomedicine, College of Pharmacy, Chongqing Medical University, Chongqing, China

**Keywords:** atherosclerosis, inflammation, interleukin-17-producing CD4^+^ T cells, macrophages, neutrophils, hyperlipidemia

## Abstract

Atherosclerosis is a chronic inflammatory disease. Interleukin-17-producing CD4^+^ T cells (Th17 cells) play important roles in the progression of atherosclerosis. However, most of the studies were focused on the advanced stage of atherosclerosis. In the current study, we investigated the roles of Th17 cells, relevant mechanisms in hyperlipidemic patients, and different stages of atherosclerotic mice. Human blood samples were collected, and percentages of Th17 cells, macrophages, and neutrophils were analyzed by flow cytometry. ApoE^−/−^ mice were fed with high-fat diet (HFD) and sacrificed at different time points to evaluate the infiltration of inflammatory cells at different stages of atherosclerosis. Furthermore, essential mechanisms of IL-17A in atherosclerotic inflammatory milieu formation were studied *in vivo* by intraperitoneal injection with monoclonal anti-murine IL-17 antibody. Our study reveals the higher percentages of Th17 cells, monocytes, and neutrophils in hyperlipidemic patients compared to healthy donors. Meanwhile, we also identify an infiltration of Th17 cells in the early stage of atherosclerosis (4 weeks after HFD), which maintains at high level until late stage of atherosclerosis (20 weeks after HFD). What is more, inflammatory cells including macrophages and neutrophils were also accumulated in atherosclerotic lesions. Neutralization of IL-17 in ApoE^−/−^ mice resulted in less infiltration of macrophages and neutrophils and smaller atherosclerotic lesions. Importantly, in accordance with what is found in the mouse model, positive correlations between Th17 cells and macrophages or neutrophils were observed in hyperlipidemic patients. In conclusion, our clinical and mouse model data together reveal a pro-atherogenic role of Th17 cells through the promotion of inflammation in hyperlipidemic conditions and different stages of atherosclerosis, which further supports the notion that IL-17 may be a therapy target for the treatment of atherosclerosis.

## Introduction

Atherosclerosis is the main cause of cardiovascular disease, which is the leading cause of mortality worldwide (1). The pathogenesis of atherosclerosis is very complicated, with a key role for immune cells and inflammation in conjunction with hyperlipidemia, especially elevated (modified) low-density lipoprotein (LDL) levels (2). Lesions of atherosclerosis contain macrophages, T cells, and other immune cells, together with cholesterol that infiltrates from the blood (3). Emerging data indicate that immune cells are affected and behave differently in a hyperlipidemic environment (4).

As part of the adaptive immune system, T cells actively participate in regulating local and systemic inflammation during atherosclerosis (5). The respective roles of T helper 1 (Th1) cells, T helper 2 (Th2) cells, and regulatory T (Treg) cells in atherosclerosis are well-established (6). Th1 cells secrete interferon-γ (IFN-γ), which promotes monocyte infiltration, enhances macrophage activation, and modulates foam-cell formation (7). Th2 cells are much less frequent in atherosclerotic plaques than Th1 cells (7) are. Th2 cells produce anti-inflammatory factors including IL-5 (8) and IL-33 (9) and play atheroprotective roles. Tregs are subdivided into two types (natural and induced) depending on their origin (10). Both natural Tregs and iTregs are important for protection against atherosclerosis, either by direct effects on T cells or through deactivation of dendritic cells (11).

Besides the T cell subsets mentioned above, the roles of IL-17-producing CD4^+^ T cells (Th17 cells) in atherosclerosis are controversial (12). Th17 cells are a new lineage of CD4^+^T cells characterized by the expression of IL-17A, IL-17F, IL-21 and IL-22, mainly IL-17A (13). IL-17 was first described as cytotoxic T lymphocyte-associated antigen 8 in 1993 and now refers to IL-17A, which is the founding member of the IL-17 family (14, 15). The expression of IL-17 was lower in non-inflammatory conditions. However, IL-17 is rapidly induced after bacterial and fungal infection and promotes leukocyte recruitment to inflammatory sites by producing chemokines and cytokines (14–16). Recently, several studies have investigated the role of IL-17 in atherosclerosis, but the results are inconsistent (12). Inhibition of IL-17A markedly reduced the atherosclerotic lesion area, maximal stenosis, and vulnerability of the lesion in apolipoprotein E knock-out (ApoE^−/−^) mice (17). However, in another study, *in vivo* administration of IL-17 in low-density lipoprotein receptor knock-out (Ldlr^−/−^) mice reduces endothelial vascular cell adhesion molecule-1 expression and vascular T cell infiltration and significantly limits atherosclerotic lesion development (18). The discrepancy of Th17 cell's roles in atherosclerosis depends on the mouse model used, the strategy used to block or supplement IL-17, or the time of the fat diet (8).

Herein we use the peripheral blood of hyperlipidemic patients and mouse model of atherosclerosis to demonstrate the dynamic changes and functions of Th17 cells at different stages of atherosclerosis in order to provide new therapy targets for the treatment of atherosclerosis.

## Materials and Methods

### Patients and Specimens

Fresh peripheral blood was obtained from 129 hyperlipidemic patients at University-Town Hospital of Chongqing Medical University. The range of ages of the hyperlipidemic patients was from 29 to 79, and the median age was 55. Among these patients, 48.1% were male and 51.9% were female. None of the patients had other metabolic diseases such as hypertension or diabetes. Peripheral blood from 110 healthy donors was used as control. The study was approved by the Ethics Committee of Chongqing Medical University. Written informed consent was obtained from each subject.

### Animals

Six-week-old male ApoE^−/−^ mice (C57BL/6 background) were purchased from Beijing Huafukang Biotechnology Co. C57BL/6 mice (wild type, WT) were obtained from Chongqing Medical University Animal Center. All mice were bred in specific pathogen-free conditions. All animal experiments were undertaken with review and approval from the Animal Ethical and Experimental Committee of Chongqing Medical University.

### Atherosclerosis Mouse Model

ApoE^−/−^ mice were fed with high-fat diet (HFD) containing 0.15% cholesterol (purchased from Medicine Ltd., China) to generate lipid-induced atherosclerosis. WT mice were fed with chow diet as control. At 4, 8, 12, 16, and 20 weeks after feeding, ApoE^−/−^ and WT mice were anesthetized and sacrificed. Blood samples were obtained and collected in Eppendorf tubes containing heparin sodium. The vasculature was perfused completely with sterile phosphate-buffered saline (PBS)–heparin sodium solution by cardiac puncture to wash out blood from the heart and all vessels. Hearts were excised and fixed in 4% paraformaldehyde. Spleen, aorta, femur, and tibia were collected in sterile PBS solution for further use.

### Flow Cytometry

Bone marrow was collected from femur and tibia and processed into single-cell suspension. Aortas were digested for 1 h at 37°C using an enzyme mixture that contained 450 U/ml collagenase I (Sigma-Aldrich, USA), 125 U/ml collagenase XI (Sigma-Aldrich, USA), 60 U/ml DNAse I (Sigma-Aldrich, USA), and 60 U/ml hyaluronidase (Sigma-Aldrich, USA) as previously reported (19). All samples were processed into single-cell suspension. These cells were stained with fluorescence-labeled antibodies of CD45, CD3, F4/80, CD14, Ly6G, CD66b, CD8, and γδ T for 30 min at 4°C. For intracellular staining of IL-17, the cells were stimulated for 6 h with cell activation cocktail with Brefeldin A (Biolegend, USA). Intracellular cytokine staining was performed after the cells were fixed and permeabilized with fixation/permeabilization buffer (eBioscience, USA) for 20 min. Supplementary Table 1 shows detailed information of the antibodies used in flow cytometry.

### Blood Lipid Measurement

Blood lipids including total cholesterol (TC), total triglyceride (TG), low-density lipoprotein-cholesterol (LDL-C), and high-density lipoprotein-cholesterol (HDL-C) were measured with corresponding assay kit (Mindray, China) using biochemical analyzer (Mindray, China).

### Assessment of Atherosclerotic Lesion

Frozen sections of aortic sinuses were stained with Oil Red O (ORO; Solarbio, China). Total lesion areas defined as intimal atherosclerotic areas and lesion lipid accumulation areas identified by ORO-stained areas were measured using ImageJ. Paraffin-embedded hearts were cut into 6–8-μm-thick slides for hematoxylin–eosin staining and Masson's staining. The necrotic core areas and collagen in atherosclerotic lesions were measured using ImageJ.

### *In vivo* IL-17 Neutralization

ApoE ^−/−^ mice were fed with HFD. At 2 weeks later, 50 μg of mouse monoclonal anti-murine IL-17 neutralizing antibody (R&D Systems, USA) was intraperitoneally injected into mice twice a week for 4 weeks, while mice that received isotype control antibody (R&D Systems, USA) or PBS served as control. Then, the mice were sacrificed, and samples were collected as detailed above.

### Statistical Analysis

Results are expressed as mean ± SEM. Student's test was generally used to analyze the differences between the two groups. Correlations between parameters were assessed using Pearson correlation analysis and linear regression analysis as appropriate. Graphpad was used for all statistical analysis. All data were analyzed using two-tailed tests, and *p* < 0.05 was considered statistically significant.

## Results

### The Percentage of Th17 Cell Is Much Higher in Peripheral Blood of Hyperlipidemic Patients Than That of Healthy Donors

**Figure 1:**
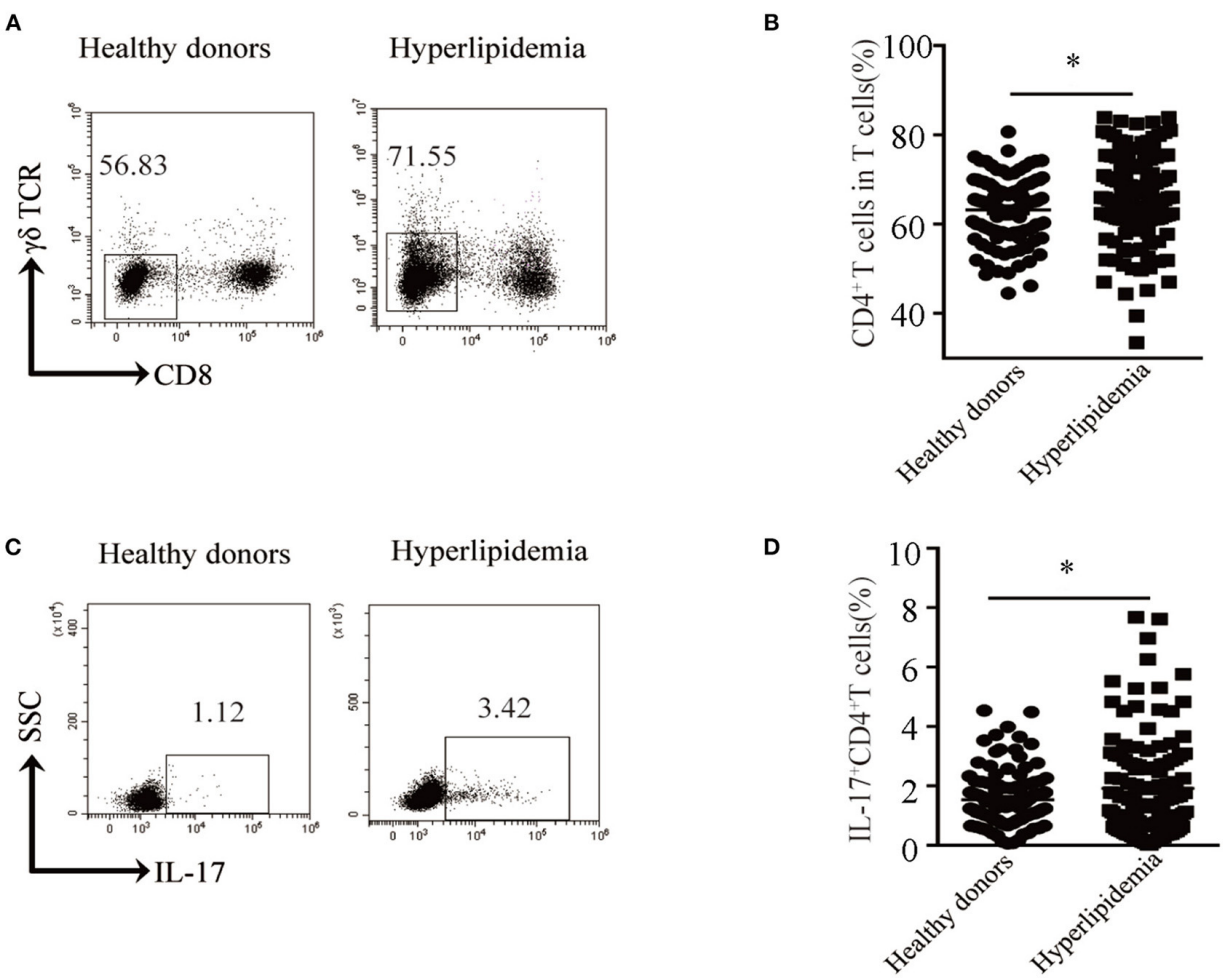
Th17 cell percentage in peripheral blood of hyperlipidemic patients. **(A)** Dot plots of surface molecule staining for CD8^−^γδTCR^−^ T cells (i.e., CD4^+^ T cells) gating on CD45^+^CD3^+^ cells. **(B)** CD4^+^ T cell percentage in CD45^+^CD3^+^ T cells. Cumulative results from 129 patients with hyperlipidemia and 110 healthy donors are shown. **(C)** Dot plots of intracellular staining of IL-17 in CD4^+^ T cell. **(D)** Th17 cell percentage in CD4^+^T cells. Each dot in **(C,D)** represents one patient. *p < 0.05.

Hyperlipidemia is one of the main risk factors of atherosclerosis. Atherosclerosis is a chronic inflammatory disease and is initiated mainly in response to endogenously modified lipoproteins, particularly oxidized low-density lipoprotein, which stimulates both the innate and adaptive immune responses (8). Given so, we first assessed the distribution of CD4^+^ T cells in the peripheral blood of hyperlipidemic patients. We found that patients with hyperlipidemia (*n* = 129) showed a higher CD4^+^ T cell percentage than that of healthy donors (*n* = 110) (Figures 1A,B). Furthermore, we assessed the production of IL-17 in CD4^+^ T cells. Notably, patients with hyperlipidemia showed a higher Th17 cell percentage than that of healthy donors (Figures 1C,D). As most of the atherosclerosis cases resulted from hyperlipidemia, these results indicate that Th17 cell may play a pivotal role in early atherosclerosis.

### Th17 Cells Are Enriched in a Mouse Model of Atherosclerosis

To identify the exact roles of Th17 cells in all stages of atherosclerosis, we established an experimental atherosclerosis model by feeding ApoE^−/−^ mice with HFD. The mice were sacrificed at 4, 8, 12, 16, and 20 weeks after HFD. The blood lipid levels (including TC, TG, HDL-C, and LDL-C) of ApoE^−/−^ mice were significantly higher than those of WT mice fed with chow diet (Supplementary Figures 1A–D). Oil red O staining showed that plaques appeared on the aortic roots of ApoE^−/−^ mice after 4 weeks of HFD, and the area of plaques gradually increased as the feeding time increased (Supplementary Figures 2A,B). The collagen fiber contents (Supplementary Figures 2C,D) and necrotic core (Supplementary Figures 2E,F) area showed the same trend. These results confirmed the successful construction of an atherosclerosis mouse model.

**Figure 2:**
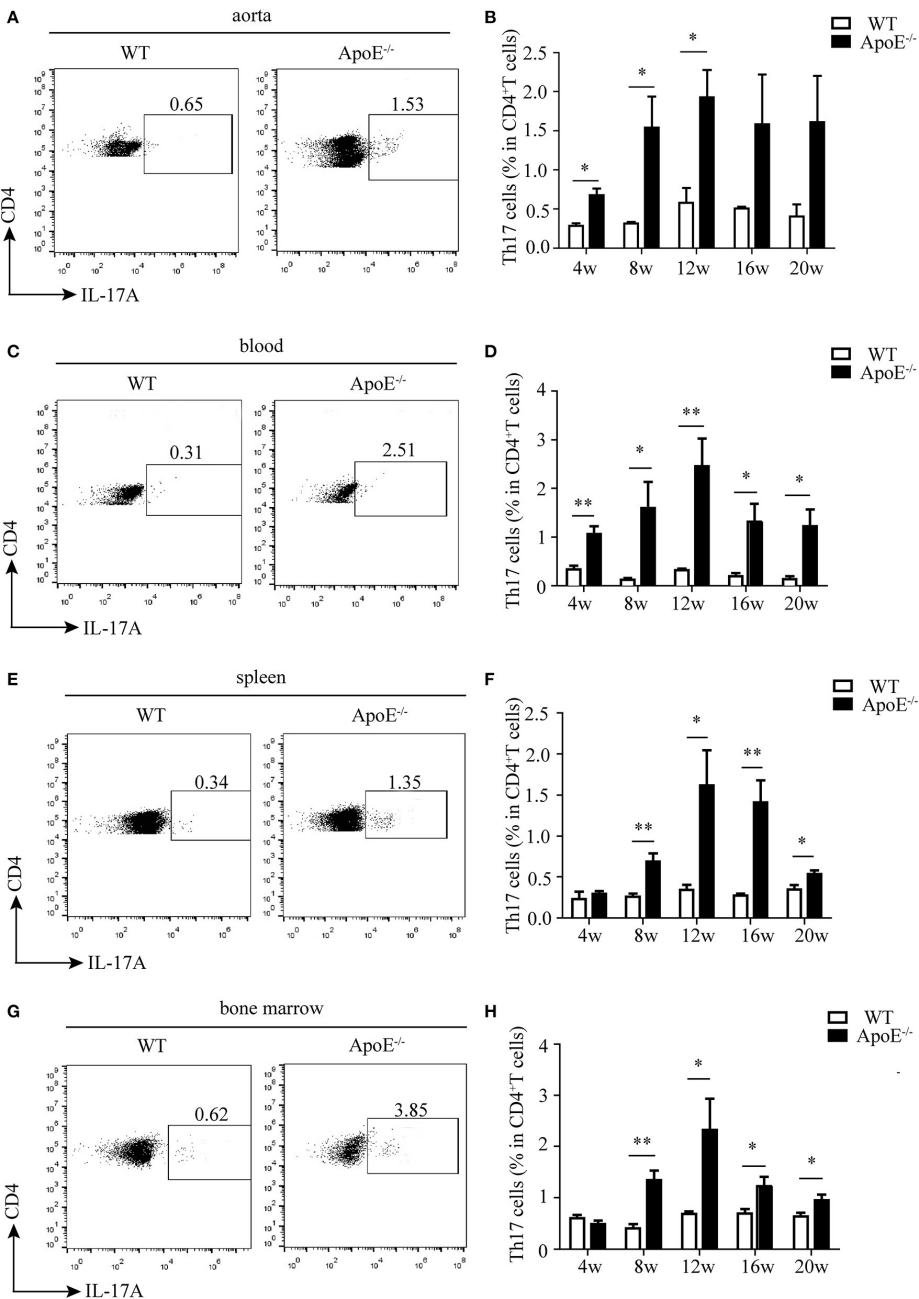
Ratio of Th17 cells in CD3^+^ T cells in different tissues and organs. Representative dot plots of intracellular staining of IL-17 gating on CD4^+^ T cells in **(A)** aorta, **(C)** blood, **(E)** spleen, and **(G)** bone marrow of wild-type (WT) and ApoE^−/−^ mice fed with high-fat diet (HFD) for 12 weeks. Th17 cell percentage in **(B)** aorta, **(D)** blood, **(F)** spleen, and **(H)** bone marrow of WT and ApoE^−/−^ mice fed with HFD at different time points (4–20 weeks). Each group consisted of six mice. *p < 0.05, **p < 0.01.

Then, we analyzed the percentage of CD4^+^T cells in different stages of atherosclerosis. The proportion of CD4^+^T in total CD3^+^T cells significantly increased in the aorta, blood, and spleen of ApoE^−/−^ mice with HFD compared to WT mice with chow diet, while no difference was observed in the bone marrow (Supplementary Figures 3A–H). What is more, Th17 cell percentage in the aorta of ApoE^−/−^ mice was much higher than that of WT mice, which reached the highest level at 12 weeks after HFD (Figures 2A,B). The same results were obtained in the blood, spleen, and bone marrow of ApoE^−/−^ mice (Figures 2C–H). These results indicate that Th17 cell may play important roles in the whole stages of atherosclerosis.

### Inflammatory Milieu Is Observed in Atherosclerotic Lesions of ApoE^–/–^ Mice

**Figure 3:**
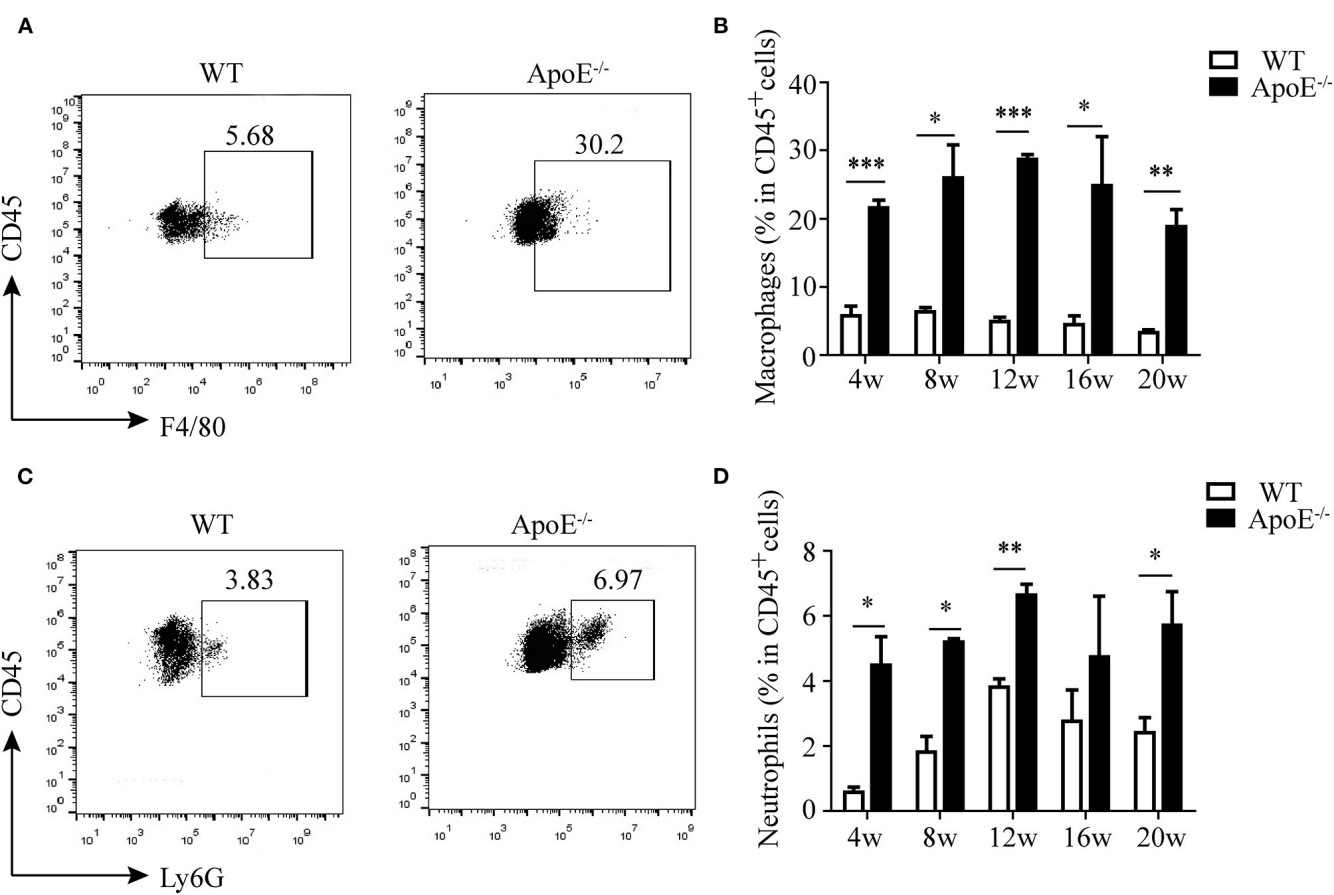
Infiltration of innate immune cells in the aorta. **(A)** Representative dot plots of F4/80^+^ macrophages gating on CD45^+^ leukocytes in the aorta of wild-type (WT) and ApoE^−/−^ mice fed with high-fat diet (HFD) for 12 weeks. **(B)** Percentage of macrophages in the aorta of WT and ApoE^−/−^ mice fed with HFD at different time points (4–20 weeks). **(C)** Representative dot plots of Ly6G^+^ neutrophil gating on CD45+ leukocytes in the aorta of WT and ApoE^−/−^ mice fed with HFD for 12 weeks. **(D)** Percentage of neutrophils in the aorta of WT and ApoE^−/−^ mice fed with HFD at different time points (4–20 weeks). Each group consisted of six mice. *p < 0.05, **p < 0.01, ***p < 0.001.

It is well-recognized that atherosclerosis is an inflammatory disease with innate and adaptive immune cells infiltrating in the vessel wall (8). Thus, we detected the infiltration of monocytes/macrophages and neutrophils besides CD4^+^ T cells. We found that monocytes/macrophages aggravated in the aorta of ApoE^−/−^ mice since 4 weeks after HFD until 20 weeks (Figures 3A,B). The same results were obtained as to the infiltration of neutrophils (Figures 3C,D).

### Neutralization of IL-17 Alleviates Atherosclerotic Lesions Through Decreasing the Cellularity in ApoE^–/–^ Mice

IL-17 can induce the release of chemokines such as C-X-C motif chemokine ligand (CXCL) 1, CXCL2, and CXCL8 by endothelial cells and vascular smooth muscle cells (20). These chemokines can recruit macrophages and neutrophils to the inflammatory sites. Thus, we wonder if IL-17 can recruit macrophages and neutrophils to atherosclerotic lesions. To test this hypothesis, the anti-IL-17 monoclonal antibody was intraperitoneally injected into ApoE^−/−^ mice, and the infiltration of macrophages and neutrophils and atherosclerotic formation were detected.

To evaluate the depletion of IL-17, the mice were sacrificed 4 weeks after anti-IL-17 antibody injection. As shown in Supplementary Figure 4, the ratios of Th17 cells in the aorta, blood, spleen, and bone marrow were significantly reduced in the anti-IL-17 group compared to the control immunoglobulin G group and PBS group, which confirmed the successful neutralization of IL-17.

**Figure 4:**
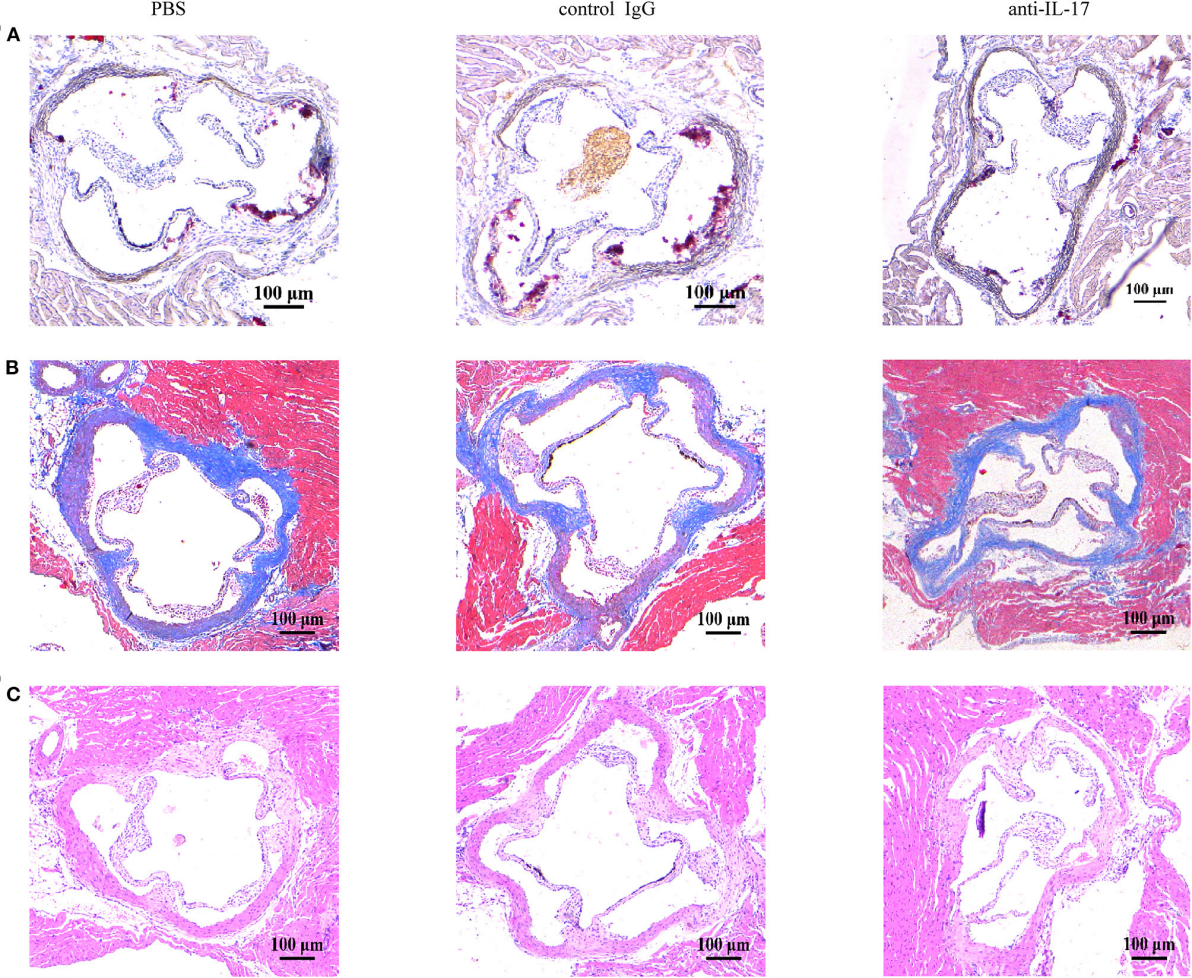
The severity of atherosclerosis after IL-17 neutralization. Representative images of aortic roots stained with **(A)** Oil Red O stain, **(B)** Masson trichrome stain, and **(C)** hematoxylin–eosin stain.

**Figure 5:**
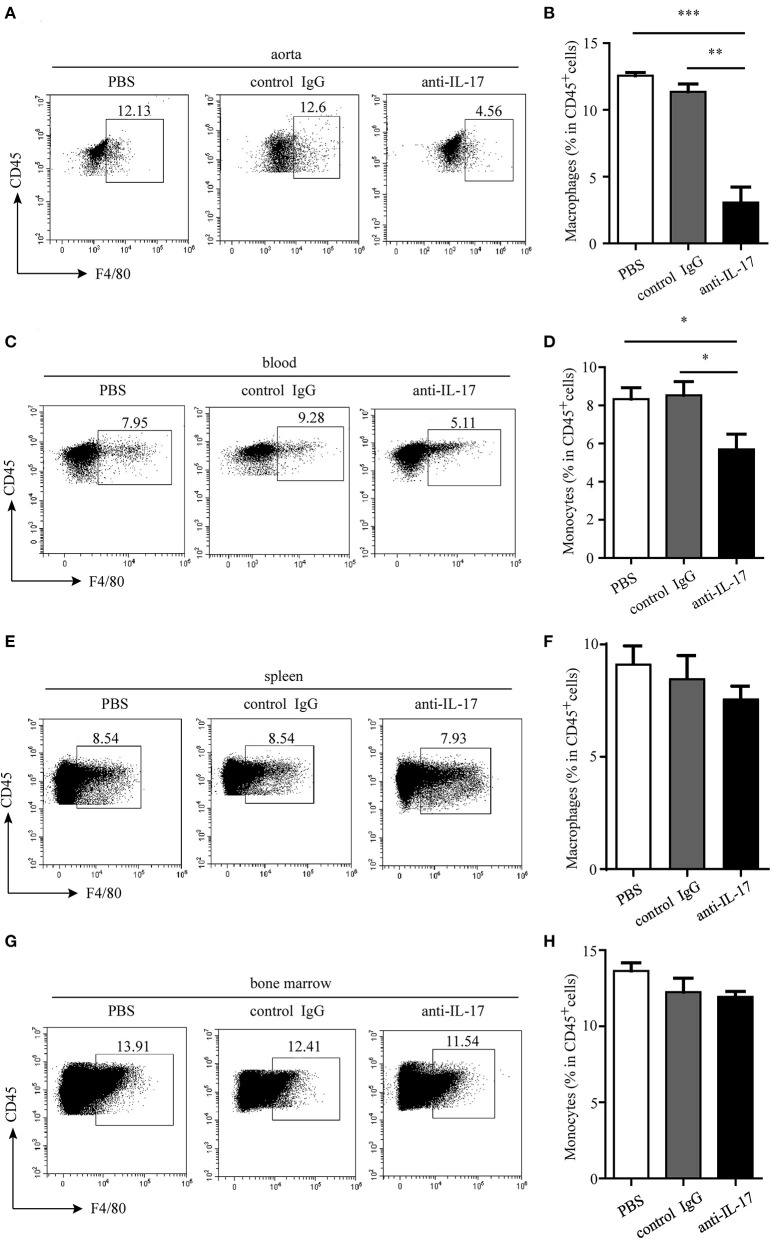
Monocyte/macrophage infiltration in different organs after IL-17 blocking. Representative dot plots of monocyte/macrophage gating on CD45^+^ leukocytes and quantitative analysis of monocyte/macrophage percentages in **(A,B)** aorta, **(C,D)** blood, **(E,F)** spleen, and **(G,H)** bone marrow. *p < 0.05, **p < 0.01, ***p < 0.001.

**Figure 6:**
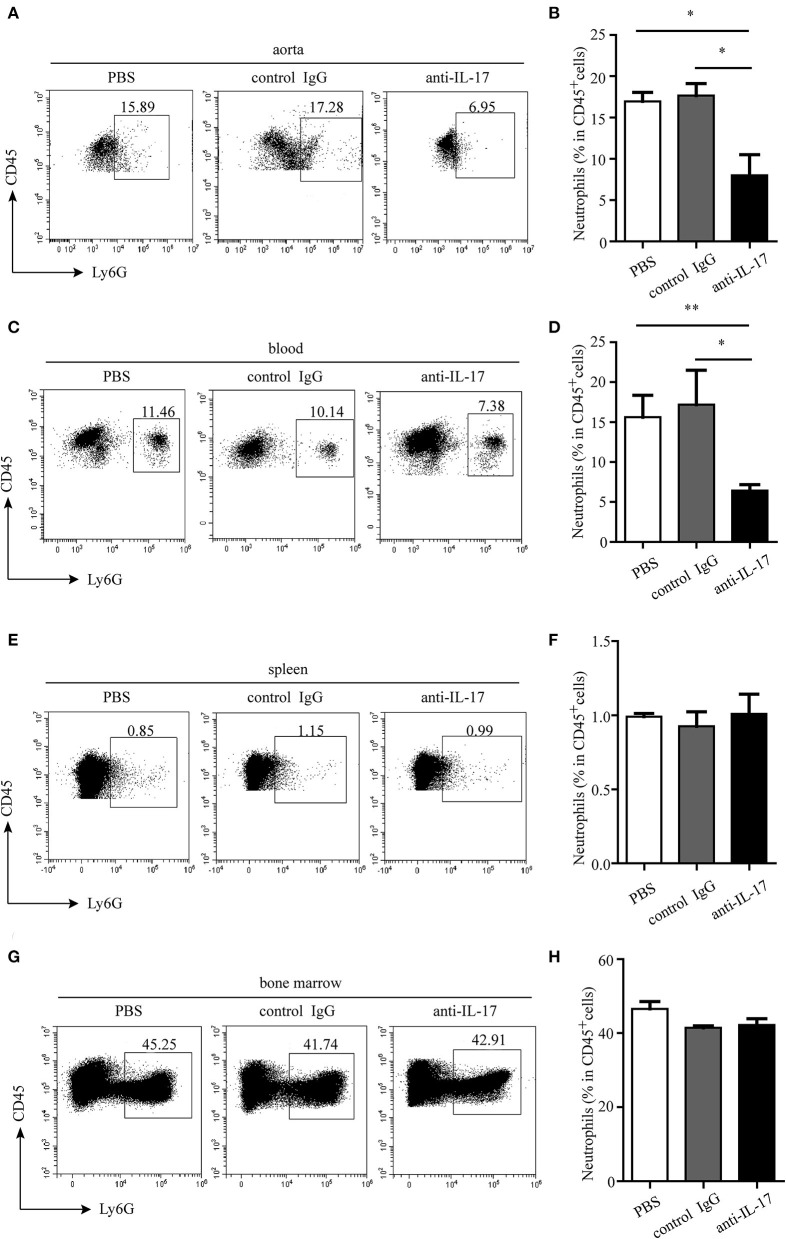
Neutrophil infiltration in different organs after IL-17 blocking. Representative dot plots of neutrophil gating on CD45^+^ leukocytes and quantitative analysis of neutrophil percentages in **(A,B)** aorta, **(C,D)** blood, **(E,F)** spleen, and **(G,H)** bone marrow. *p < 0.05, **p < 0.01.

The atherosclerotic plaque formation was alleviated in mice deficient in IL-17 as the lesion size, collagen contents, and necrotic core all decreased (Figures 4A–C). Monocytes/macrophages were significantly reduced in the aorta and blood of mice injected with anti-IL-17 antibody (Figures 5A–D), while no obvious difference was found in the spleen or bone marrow among the different groups (Figures 5E–H). The same results were obtained as to the infiltration of neutrophils (Figures 6A–H). These results imply that IL-17 may promote the migration of innate immune cells to atherosclerotic lesions rather than influence their production in the bone marrow. As the result of macrophage and neutrophil infiltration, atherosclerotic plaque formation was exacerbated further.

### Th17 Cells Are Positively Correlated With Monocytes and Neutrophils in Hyperlipidemic Patients

**Figure 7:**
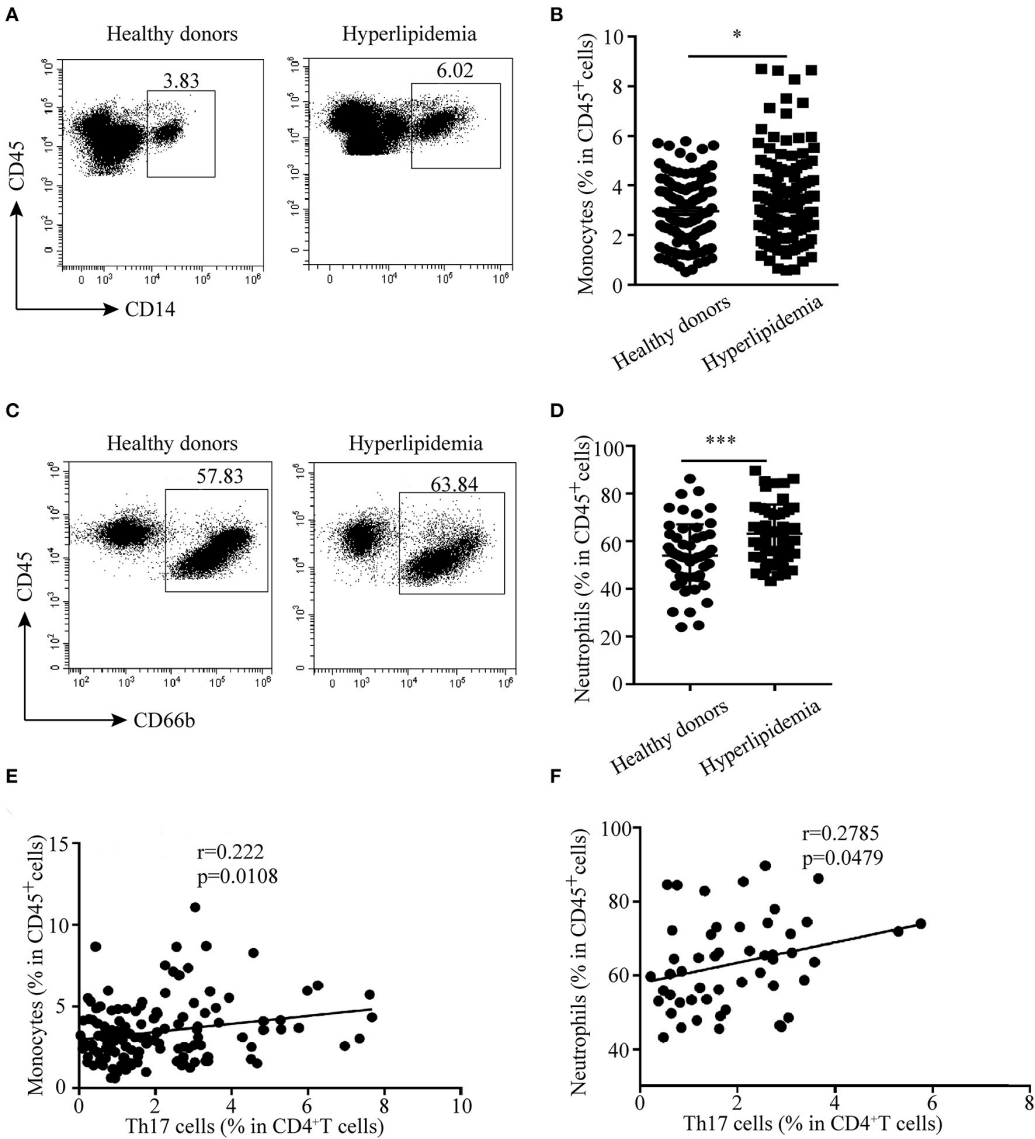
Correlation between Th17 cells and monocytes and neutrophils in hyperlipidemic patients. **(A,B)** Percentages of monocytes (CD45^+^CD14^+^ cells) in human peripheral blood. **(C,D)** Neutrophil (CD45^+^CD66b^+^cells) percentage in human peripheral blood. **(E)** Correlation between Th17 cells and monocytes (*n* = 129). **(F)** Correlation between Th17 cells and neutrophils (*n* = 51). *p < 0.05, ***p < 0.001.

The relationship between Th17 cells and other innate immune cells in atherosclerotic mice made us wonder whether there was the same phenomenon in human hyperlipidemia. Then, the distribution of monocytes and neutrophils in peripheral blood of hyperlipidemic patients was analyzed by flow cytometry. The results showed that the percentages of monocytes and neutrophils were much higher in hyperlipidemic patients than those in healthy donors (Figures 7A–D). Further analysis revealed that Th17 cells had a positive correlation with monocytes and neutrophils, respectively (Figures 7E,F). The results were in accordance with that observed in IL-17-deficient mice, which implies a regulatory role of Th17 cells in the early stage of atherogenesis.

## Discussion

Our findings reveal that Th17 cells increased since early atherosclerosis, and the neutralization of IL-17 results in smaller atheroma plaque formation in ApoE^−/−^ mice fed with HFD. We identify the migration of macrophages and neutrophils in atherosclerotic lesions regulated by Th17 cells as the mechanism potentially responsible for the observed phenomenon. Our clinical data support the concept because there is a positive correlation between Th17 cells and monocytes or neutrophils in hyperlipidemic patients. These observations illustrate that Th17 cells play pivotal roles in not only the genesis but also the development of atherosclerosis.

Atherogenesis is initiated by the accumulation of apolipoprotein-B-containing lipoproteins in the intima at regions of disturbed blood flow in medium-sized arteries (21). This event triggers the recruitment of monocytes/macrophages, the most abundant immune cells present in atherosclerotic lesions, as well as cells of adaptive immune response such as T lymphocytes (22). In patients with hyperlipidemia (mainly high levels of TC and LDL-C), we found an increased percentage of Th17 cells in peripheral blood compared to healthy donors. As hyperlipidemia is an initiation state of atherosclerosis, we speculate that Th17 cells may infiltrate in the early stage of atherosclerosis. In accordance with this, significantly higher infiltration of Th17 cells was observed in the aorta, blood, and spleen of ApoE^−/−^ mice at the early stage of atherogenesis (4 weeks after HFD). Because most of the recent studies in regard to the role of Th17 cells are focused on the advanced stage of atherosclerosis, we also detect the infiltration of Th17 cells in ApoE^−/−^ mice fed with HFD at different time points (i.e., 8, 12, 16, and 20 weeks after HFD). The increased infiltration of Th17 cells not only exists at the early stage of atherosclerosis but is also maintained at high levels until the late stage of atherosclerosis. These data indicate that Th17 cells may be involved in the initiation and development process of atherosclerosis.

As the main product of Th17 cell, IL-17A plays important roles in autoimmune diseases such as multiple sclerosis, inflammatory bowel disease, and arthritis (23). It affects local inflammation in several ways. IL-17A induces the production of cytokines IL-6 and IL-8 as well as chemokines C-C motif chemokine ligand (CCL) 5, CCL2, and CXCL 1 by a variety of cells, including endothelial and vascular smooth muscle cells, fibroblasts, and epithelial cells (24). These cytokines and chemokines then accelerate the recruitment of leukocytes to atherosclerotic vessels (18). In support of this notion, we detected increased numbers of macrophages and neutrophils in the aorta of ApoE^−/−^ mice fed with HFD. Moreover, intraperitoneal injection of anti-IL-17A antibody *in vivo* resulted in the diminished infiltration of monocytes/macrophages and neutrophils in the aorta, blood, and spleen of ApoE^−/−^ mice, while the macrophages and neutrophils in the bone marrow were not affected. What is important is that our clinical data also implied a positive relationship between Th17 cells and monocytes or neutrophils. These data suggest that Th17 cells may promote the progression of atherosclerosis via enhancing the mobilization, instead of the genesis, of inflammatory cells. However, further studies are needed to uncover the specific mechanisms about how macrophages and neutrophils are regulated by Th17 cells in atherosclerosis.

Collectively, using the clinical blood samples of hyperlipidemic patients and an atherosclerotic mouse model, we demonstrate a pro-atherogenic effect of IL-17 from early to late stages of atherosclerosis. In hyperlipidemic conditions, CD4^+^ T cells are activated and produce inflammatory cytokines, especially IL-17, in the aorta, blood, spleen, and bone marrow, which induces the migration of macrophages and neutrophils to atherosclerotic lesions and exacerbates the development of atherosclerosis. Understanding how Th17 cells are regulated in hyperlipidemic conditions and in the different stages of atherosclerosis and the interactions among immune cells in atheromatous plaque are of utmost importance to identify potential therapeutic targets to prevent or stabilize the disease process. Our findings further support the notion that IL-17 may be a therapy target for the treatment of atherosclerosis.

## Data Availability Statement

The original contributions presented in the study are included in the article/Supplementary Material, further inquiries can be directed to the corresponding authors.

## Ethics Statement

The studies involving human participants were reviewed and approved by Ethics Committee of Chongqing Medical University. Written informed consent to participate in this study was provided by the participants' legal guardian/next of kin. The animal study was reviewed and approved by Animal Ethical and Experimental Committee of Chongqing Medical University.

## Author Contributions

TW and CY: conception and design, data analysis, and manuscript revision. YW: experiment conduction, data analysis, and drafting the manuscript. WL: blood samples and clinical data collection. TZ and YZ: experiment conduction. TD, ZYa, ZYu, LM, and RY: technical support and editing. All authors contributed to the article and approved the submitted version.

## Conflict of Interest

The authors declare that the research was conducted in the absence of any commercial or financial relationships that could be construed as a potential conflict of interest.
