# Supplementary material for: Interleukin-17-Producing CD4+ T Cells Promote Inflammatory Response and Foster Disease Progression in Hyperlipidemic Patients and Atherosclerotic Mice
Source: Front Cardiovasc Med. 2021 Apr 26;8:667768. doi: 10.3389/fcvm.2021.667768 (PMC8107221; doi:10.3389/fcvm.2021.667768)
Supplement: Supplementary file 1 [file Data_Sheet_1.PDF]

Supplementary Table S1. Antibodies used in flow cytometry

Anti-mouse antibodies for flow cytometry	Manufacturers
Anti-CD45-PE/Cy7	Biolegend
Anti-F4/80-FITC	Biolegend
Anti-Ly6G-Percp/Cy5.5	Biolegend
Anti-CD45-BV605	Biolegend
Anti-CD3-APC/Cy7	Biolegend
Anti-CD8b2-PE/Cy7	Biolegend
Anti-γδ T-BV650	BD Bioscience
Anti-IL-17A-APC	invitrogen
Anti-human antibodies for flow cytometry	
Anti-CD45-PE/Cy7	Biolegend
Anti-CD14-FITC	Biolegend
Anti-CD66b-FITC	Biolegend
Anti-CD3-BV650	Biolegend
Anti-CD8α-APC/Cy7	Biolegend
Anti-γδ T-PerCP/Cy5.5	Biolegend
Anti-IL-17A-Alexa Fluor 488	Biolegend

APC, allophycocyanin; APC/Cy7, allophycocyanin/cyanine 7; FITC, fluorescein isothiocyanate; PE, phycoerythrin; PE/Cy7, phycoerythrin/cyanine 7; PerCP/Cy5.5, peridin chlorophyl protein/cyanine 5.5; BV650, Brilliant Violet650TM; BV605, Brilliant Violet605TM.

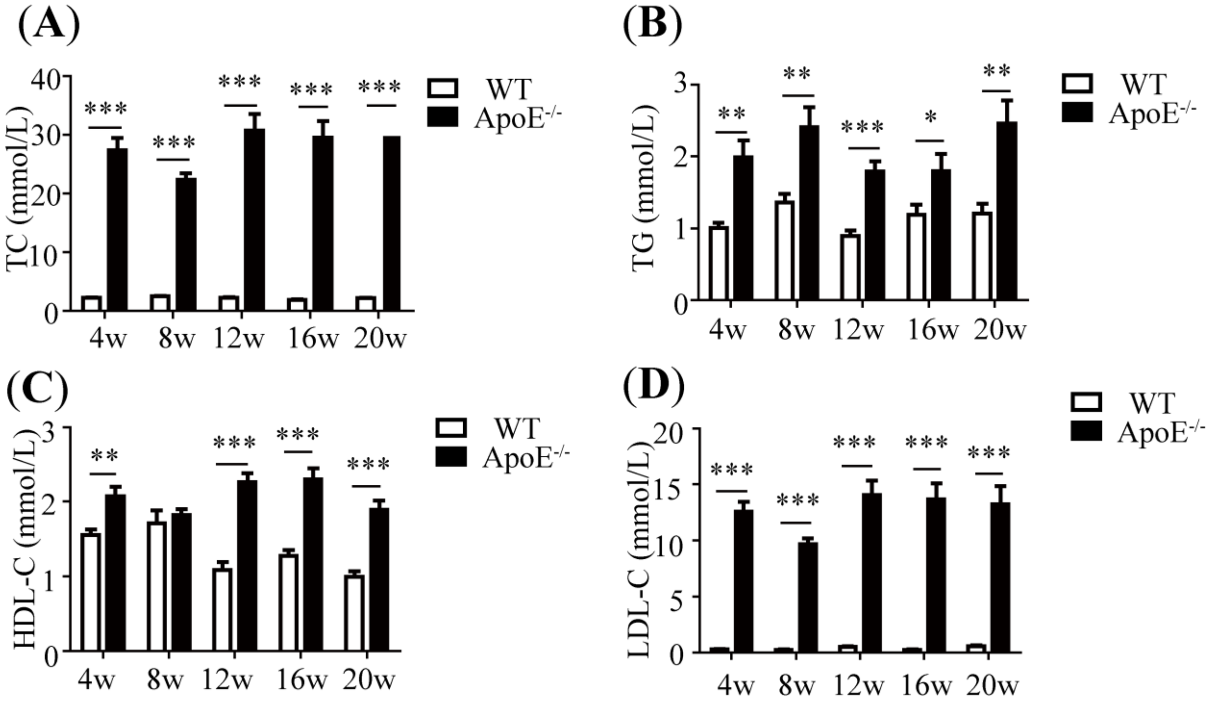

Supplementary Figure S1. Blood lipid levels of ApoE-/- and WT mice. (A) Total cholesterol (TC), (B) triglycerides (TG), (C) high-density lipoprotein cholesterol (HDL-C) and (D) low density lipoprotein cholesterol (LDL-C) levels measured by biochemical analyzer. *$p < 0.05$, **$p < 0.01$, ***$p < 0.001$.

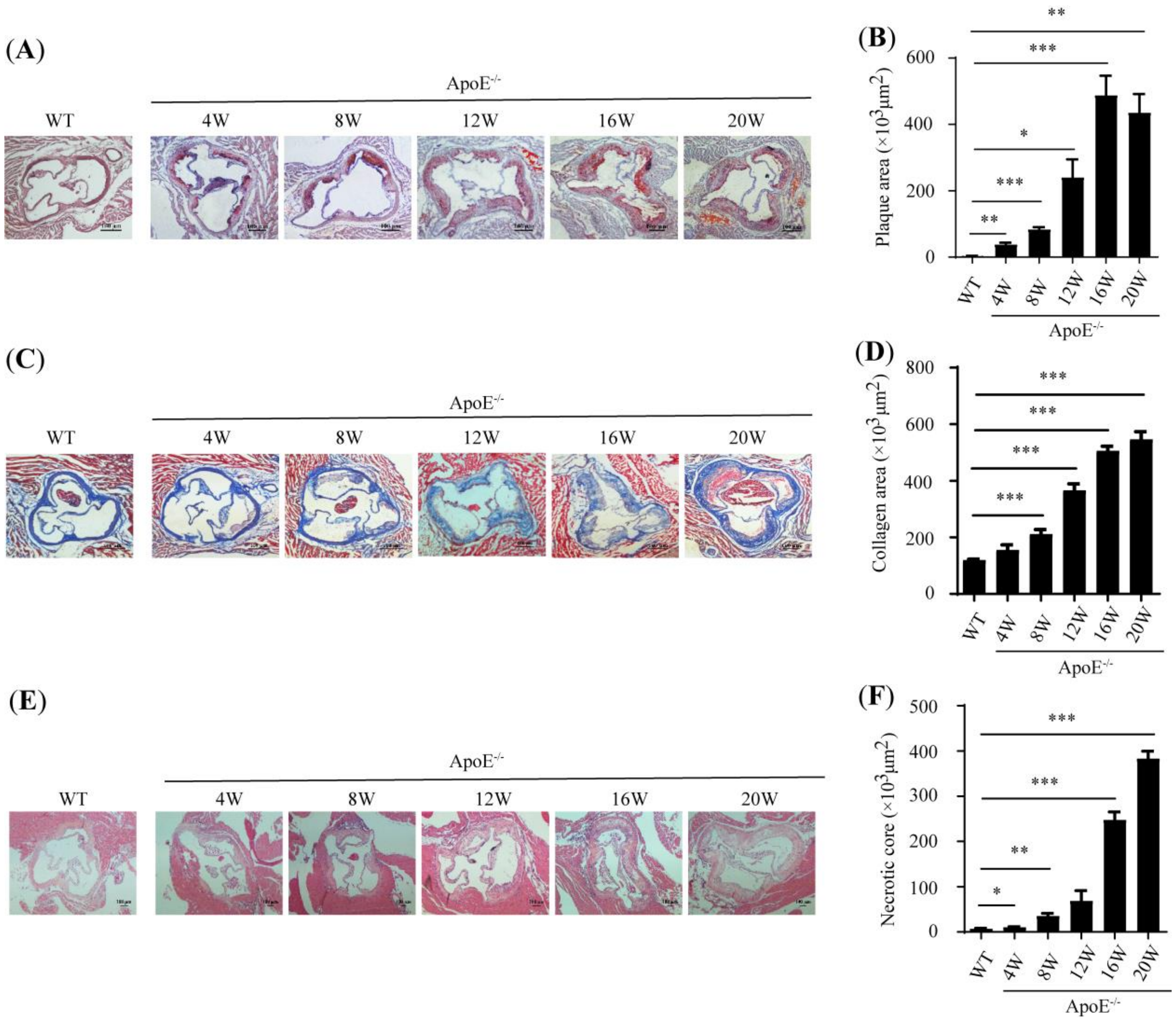

Supplementary Figure S2. Construction of atherosclerosis mouse model. (A) Oil Red O staining of mouse aortic sinus. (B) Quantification of total atherosclerotic lesion size. (C) Masson trichrome staining of mouse aortic sinus. (D) Quantification of collagen fiber contents. (E) HE-staining of mouse aortic sinus. (F) Quantification of necrotic core areas. *$p<0.05$, **$p<0.01$, ***$p<0.001$.

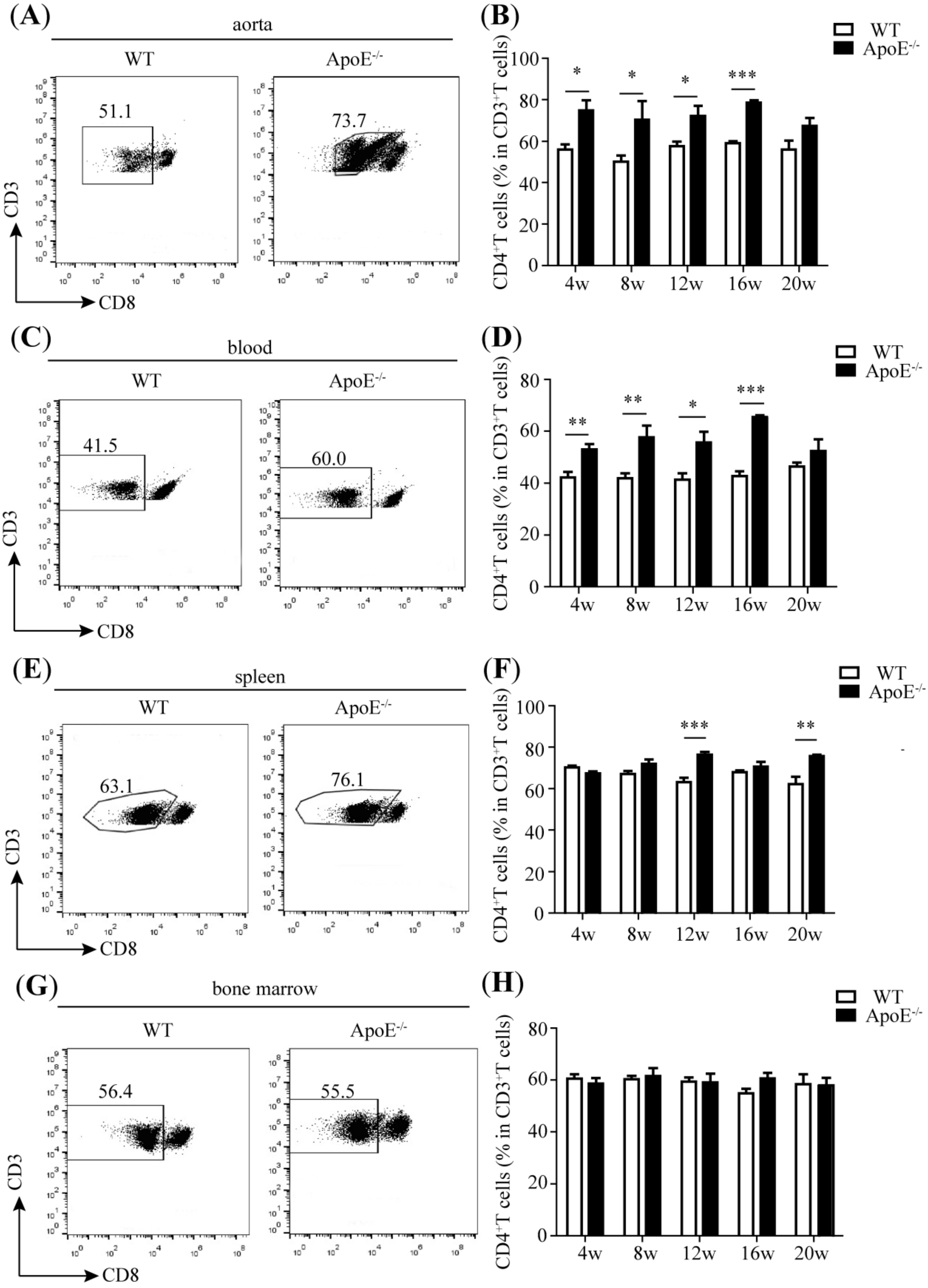

Supplementary Figure S3. The ratio of CD4+T cells in different tissues and organs. (A) Representative dot plots of CD4+T cells gating on CD3+T cells in (A) aorta, (C) blood, (E) spleen, and (G) bone marrow of WT and ApoE-/- mice fed with HFD for 12 weeks. Percentages

of $CD4^+$T cells in (B) aorta, (D) blood, (F) spleen, and (H) bone marrow of $ApoE^{-/-}$ and WT mice fed with HFD at different time points (4 weeks to 20 weeks). Each group contained 6 mice. *$p < 0.05$, **$p < 0.01$, ***$p < 0.001$.

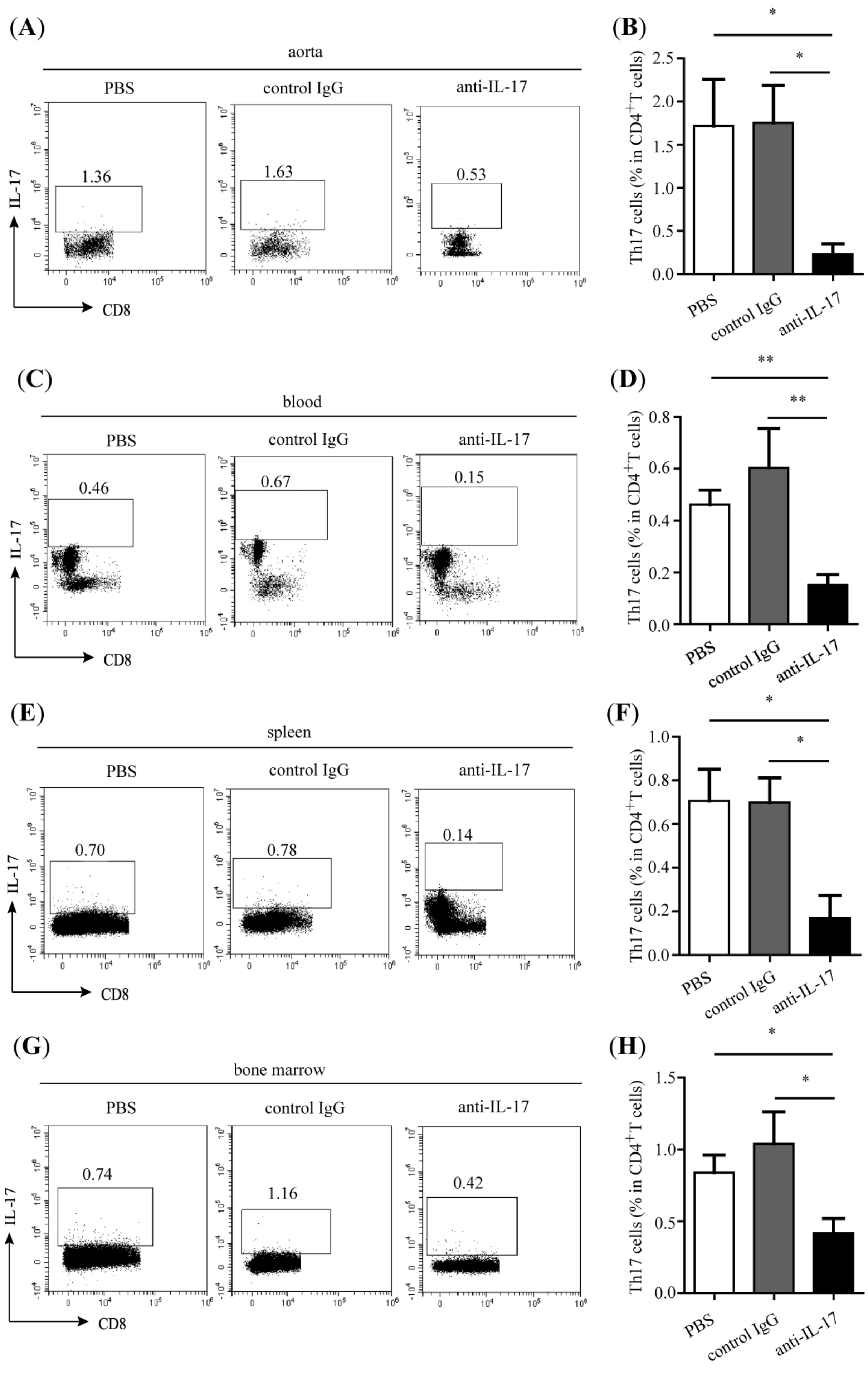
(A)
aorta
PBS
control IgG
anti-IL-17
1.36
1.63
0.53
IL-17
CD8
(B)
Th17 cells (% in CD4+T cells)
PBS
control IgG
anti-IL-17
(C)
blood
PBS
control IgG
anti-IL-17
0.46
0.67
0.15
IL-17
CD8
(D)
Th17 cells (% in CD4+T cells)
PBS
control IgG
anti-IL-17
(E)
spleen
PBS
control IgG
anti-IL-17
0.70
0.78
0.14
IL-17
CD8
(F)
Th17 cells (% in CD4+T cells)
PBS
control IgG
anti-IL-17
(G)
bone marrow
PBS
control IgG
anti-IL-17
0.74
1.16
0.42
IL-17
CD8
(H)
Th17 cells (% in CD4+T cells)
PBS
control IgG
anti-IL-17

Supplementary Figure S4. The depletion of IL-17 after anti-IL-17 antibody injection. Th17 percentage in (A-B) aorta, (C-D) blood, (E-F) spleen and (G-H) bone marrow. Each group contained 6 mice (n=6). *$p < 0.05$, **$p < 0.01$, ***$p < 0.001$.
